# DEPDC1B regulates the progression of human chordoma through UBE2T-mediated ubiquitination of BIRC5

**DOI:** 10.1038/s41419-021-04026-7

**Published:** 2021-07-30

**Authors:** Liang Wang, Liang Tang, Ruijun Xu, Junpeng Ma, Kaibing Tian, Yanbin Liu, Yanghu Lu, Zhen Wu, Xiaodong Zhu

**Affiliations:** 1 Department of Neurosurgery, Beijing Tiantan Hospital, Capital Medical University, No. 119 Nansihuan Xilu, Beijing, 100070 China; 2 Department of Orthopaedic Surgery, Tongren Hospital, Shanghai Jiao Tong University School of Medicine, No. 1111 Xianxia Road, Shanghai, 200336 China; 3 Department of Orthopaedic Surgery, Renji Hospital, Shanghai Jiao Tong University School of Medicine, No. 2000 Jiangyue Road, Shanghai, 200127 China

**Keywords:** CNS cancer, Oncogenes

## Abstract

Chordoma is a rare bone malignancy with a high rate of local recurrence and distant metastasis. Although DEP domain-containing protein 1B (DEPDC1B) is implicated in a variety of malignancies, its relationship with chordoma is unclear. In this study, the biological role and molecular mechanism of DEPDC1B in chordoma were explored. The function of DEPDC1B in chordoma cells was clarified through loss-of-function assays in vitro and in vivo. Furthermore, molecular mechanism of DEPDC1B in chordoma cells was recognized by RNA sequencing and Co-Immunoprecipitation (Co-IP) assay. The malignant behaviors of DEPDC1B knockdown chordoma cells was significantly inhibited, which was characterized by reduced proliferation, enhanced apoptosis, and hindered migration. Consistently, decreased expression of DEPDC1B suppressed tumor growth in xenograft mice. Mechanically, DEPDC1B affected the ubiquitination of baculoviral inhibitor of apoptosis repeat-containing 5 (BIRC5) through ubiquitin-conjugating enzyme E2T (UBE2T). Simultaneous downregulation of BIRC5 and DEPDC1B may exacerbate the inhibitory effects of chordoma. Moreover, BIRC5 overexpression reduced the inhibitory effects of DEPDC1B knockdown in chordoma cells. In conclusion, DEPDC1B regulates the progression of human chordoma through UBE2T-mediated ubiquitination of BIRC5, suggesting that it may be a promising candidate target with potential therapeutic value.

## Introduction

Chordoma is a rare, slow-growing primary bone malignancy that originates from primitive notochordal tissue [1, 2]. In addition, chordomas have neither obvious early symptoms nor any external manifestations and are usually diagnosed at advanced [3]. Chordomas do not respond to conventional radiotherapy or cytotoxic chemotherapy, and surgery is the primary treatment option [4, 5]. Unfortunately, the complex anatomy of the spine and the relatively large tumor volume make resection technically challenging, resulting in a high rate of local recurrence and distant metastasis [4]. As a result, traditional treatment options are far from adequate [6]. In recent years, with the vigorous development of molecular biology, molecular targeted therapy is gradually being applied. For example, the nuclear expression of brachyury, a key transcription factor for notochord development, can be used as a classic diagnostic marker for chordoma [7, 8]. Moreover, a variety of potential molecular targets for chordoma were identified, such as platelet-derived growth factor receptor β, PI3K/mTOR, epidermal growth factor receptor (EGFR), vascular endothelial growth factor (VEGF), and tyrosine kinase. However, the objective response of these molecular targeting drugs is not satisfactory [9]. Therefore, identification of new potential molecular targets is essential for patients with chordoma.

The DEP Domain-Containing Protein 1B (DEPDC1B) is located on human chromosome 5q12 and encodes a 61 kDa protein consisting of 529 amino acids, which includes an N-terminal DEP domain and a C-terminal Rho-GAP (GTPase activating protein)-like domain [10]. The DEP domain is a spherical domain of about 90 amino acids, which plays a role in the localization of dielectric membranes [11]. The Rho-GAP domain is involved in Rho GTPase signal transduction, such as RAC, Cdc42, and Rho, regulating cell movement, growth, differentiation, cytoskeleton reorganization, and cell cycle process [12]. The membrane binding of DEP domain enables DEPDC1B to interact with G-protein-coupled receptors and membrane phospholipids required for Wnt signal transduction [13]. Furthermore, DEPDC1B can coordinate debonding events and cell cycle progression during mitosis [14]. Figeac et al. supported that DEPDC1B is a key regulator of mouse and human myoblast proliferation [15]. In addition, DEPDC1B is required for a variety of malignancies, such as breast cancer [11, 16], non-small cell lung cancer [17], oral cancer [18], prostate cancer [19], melanoma [20], and glioblastoma [21]. In pancreatic cancer or prostate cancer, DEPDC1B could promote migration and invasion through Rac1/PAK1 signaling [22, 23]. In bladder cancer, DEPDC1B is a tumor promotor through targeting SHC1 [24]. Accordingly, we found that DEPDC1B played a promoting role in the progression of a variety of cancers. Because our research group is dedicated to the investigation of chordoma, we wondered the role of DEPDC1B in chordoma. Therefore, the biological function and potential molecular mechanism of DEPDC1B in chordoma were explored in this study.

Our study showed that the malignant behavior of the chordoma cells after DEDDC1B knockdown was significantly inhibited. Mechanically, DEPDC1B affected the ubiquitination of baculoviral inhibitor of BIRC5 through UBE2T. Simultaneous downregulation of BIRC5 and DEPDC1B may exacerbate the inhibitory effects of chordoma. Meanwhile, BIRC5 overexpression reduced the inhibitory effects of DEPDC1B knockdown in chordoma cells. Therefore, the significant breakthrough that DEPDC1B regulates the progression of human chordoma through UBE2T-mediated ubiquitination of BIRC5 may provide a valuable target for molecular therapy of chordoma patients.

## Materials and methods

### Cell culture

The human chordoma cell lines U-CH1 and MUG-Chor1 were purchased from Cell Bank of the Chinese Academy of Sciences (Shanghai, China) and maintained the atmosphere of 37 °C with 5% CO_2_. The cells were cultured in DMEM (Corning, Cat. No. 10-013-CVR) supplemented with 10% fetal bovine serum (FBS) (Ausbian, Cat. No. VS500T) and puromycin (Gibco, Cat. No. A11138-003). Notably, U-CH1 is the first confirmed chordoma cell line with cellular genetic aberrations typical of chordomas [25]. MUG-Chor1 is markedly similar to chordomas and can grow steadily, which can be served as an optimal chordoma model in vitro [26].

### Lentiviral shRNA vector construction and cell infection

First, the RNA interference (RNAi) against DEPDC1B (shDEPDC1B-1: GCTGCTAGATTGGTAACGTTT; shDEPDC1B-2: GAGGCCAATGTAGAAGAGATA; shDEPDC1B-3: CAGCTATGAAGTGTTTGGCAA) or BIRC5 (shBIRC5-1: ATGCACTTCAGACCCACTTAT; shBIRC5-2: CGGCCTTTCCTTAAAGGCCAT; shBIRC5-3: GGCCAACTGCCATCCTGGAAA) and corresponding negative control (shCtrl: 5′-TTCTCCGAACGTGTCACGT-3′) were designed and synthesized. These sequences were ligated to BR-V-108 lentivirus vectors (Shanghai Bioscienceres, Co., Ltd) that carried the green fluorescent protein (GFP), respectively. After that, the target sequences with great knockdown effects were screened out by WB. These lentiviruses were used to infect U-CH1 and MUG-Chor1 cells and viewed under a fluorescence microscope (Olympus).

### RNA isolation and qPCR

The RNA of U-CH1 and MUG-Chor1 cells was extracted with the Trizol reagent (Sigma, Cat. No. T9424-100m) and reverse-transcribed into cDNA by Hiscript QRT Supermix (Vazyme, Nanjing, China, Cat. No. R123-01). Subsequently, the mixed reaction solution composed of cDNA, corresponding primers (Table S1) and SYBR premix (Vazyme) was performed to qPCR. Finally, GAPDH was used as an internal reference and the relative mRNA levels were estimated by 2^−ΔΔCt^.

### Western blotting (WB) analysis and co-immunoprecipitation (Co-IP) assay

After the U-CH1 and MUG-Chor1 cells were lysed, the protein was obtained and the concentration was measured by BCA protein detection kit (Thermo Fisher Scientific, California, USA). The protein with equal amounts was subjected to SDS-PAGE, transferred to nitrocellulose membranes and incubated with primary antibodies (Table S2) overnight at 4 °C. After washed by TBST, the membranes were probed with secondary antibodies. Finally, Millipore Immobilon Western Chemiluminescent HRP Substrote kit (Millipore, Cat. No. RPN2232) was used to color rendering and Chemiluminescent imager (GE, Cat. No. AI600) observation. The protein–protein interaction was analyzed by Co-IP assay and the experimental procedures were performed as previously described [27].

### MTT cell proliferation assay

The U-CH1 and MUG-Chor1 cells were inoculated at a density of 1000 cells/well and cultured overnight on a 96-well plate. On the next day, 20 μL of 5 mg/mL MTT (Genview, Cat. No. JT343) was added, the culture medium was completely absorbed 4 h later. After 100 μL DMSO was added to dissolved Formazan and crystallized. The absorption value was determined at 490 nm by enzyme micro-plate reader (Biotek Cat. No. Elx800) for 5 consecutive days.

### Cell counting assay

The MUG-Chor1 cells were cultured on a 96-well plate with a density of 2000 cells per well. After that, the cells were counted by Celigo (Nexcelom) every day for 5 days. Accordingly, the cell proliferation curve was drawn by counting the number of GFP cells in each scanning orifice plate.

### Cell clone formation assay

The MUG-Chor1 cells were inoculated at a density of 1000 cells/well on a 6-well plate for 14 days and washed with PBS buffer. After that, the cells were fixed by 4% paraformaldehyde (SIGMA, Cat. No. P6148) of 1 mL for 60 min, stained with 500 μL Giemsa (Tripod biotechnology, Cat. No. KGA229) for 20 min, washed and dried by ddH_2_O. Finally, the cells clone was photographed and counted.

### Cell apoptosis analysis by flow cytometry (FACS)

After the U-CH1 and MUG-Chor1 cells were continuously cultured in 6-well plates for 7 days, the cell precipitates were washed by precooled D-Hanks (pH = 7.2–7.4) and 1× buffer solution in turn. Subsequently, the resuspension cells were precipitated and stained with Annexin V-APC (eBioscience, Cat. No. 88-8007-74) for 10–15 min. Finally, the apoptosis rate was analyzed and calculated by flow cytometry (Millipore, Cat. No. IX73).

### Human apoptosis antibody array

The concentrations of 43 human apoptotic markers in the MUG-Chor1 cells (with or without knockdown of DEPDC1B) were detected simultaneously using human apoptosis antibody array-membrane (Abcam, Cat. No. ab134001). After the cell protein was obtained, the product instructions were followed to detect the differential expression of the groups of shCtrl and shDEPDC1B.

### Wound-healing assay

U-CH1 and MUG-Chor1 cells were cultured in the 96-well plate with a density of 5 × 10^4^ cells/well. After the low concentration of serum medium was replaced the next day, the scratches were formed by nudging upward at the center of the lower end of the 96-well plate with a scratch meter. At 0, 24, and 48 h of cell migration, the width of scratch area was measured and the migration ability was analyzed.

### Transwell assay

U-CH1 and MUG-Chor1 cells with a density of 5 × 10^4^ cells/well were incubated in the well-hydrated chamber (3422 corning). The inner chamber contained 100 μL of serum-free medium and the external chamber contained 600 μL 30% FBS. After the cell suspension was diluted with serum-free medium, the cells were added to each chamber for 24 h cultivation. After the migrating cells were fixed with 4% formaldehyde, stained with Giemsa. Finally, the cells were observed under the fluorescence microscope and photographed to estimate the migration capacity.

### Xenograft mouse tumor model

The 4-week-old female BALB/c nude mice (Lingchang Biotechnology, Shanghai, China) were divided into two groups (shDEPDC1B and shCtrl). After the MUG-Chor1 cells (with or without knockdown of DEPDC1B) were digested by trypsin, the concentration of 1E + 7 cells/ mL was maintained. Tumor growth was observed after subcutaneous injection of 200 μL of cells into the right forearm of mice for 5–7 days. Subsequently, the anesthetized mice were intraperitoneally injected with 0.7% pentobarbital sodium at 10 μL/g and placed in a living body imager for imaging and data preservation. In addition, tumor size and mice weight were measured every other day. After 21 days, the mice were euthanized and the tumors were weighed and photographed. Finally, the tumor tissues were extracted from mice and incubated with antibody Ki67 (Table S2) for IHC staining. All procedures involving mice and experimental protocols were approved by the Institutional Animal Care and Use Committees of Shanghai Jiao Tong University School of Medicine.

### RNA sequencing

Affymetrix human GeneChip PrimeView combined with Affymetrix Scanner 3000 was performed to elaborate the molecular mechanism. Accordingly, the volcano plot and hierarchical clustering of the MUG-Chor1 cells (with or without knockdown of DEPDC1B) were presented by the differentially expressed genes (DEGs) with criterion of |Fold Change| ≥ 2 and false discovery rate (FDR) < 0.05. Furthermore, the significant enrichment of DEGs in canonical pathway, and diseases or functions were investigated based on ingenuity pathway analysis (IPA).

### Statistical analysis

Statistical analyses were accomplished by SPSS 19.0 with GraphPad Prism 8.0 software, the data were presented as the mean ± standard deviation. The independent Student’s *t* test was used to analyze the statistical significance between different groups and *P* < 0.05 was considered statistically significant.

## Results

### Knockdown of DEPDC1B inhibits the malignant behaviors of chordoma cells in vitro

**Fig. 1:**
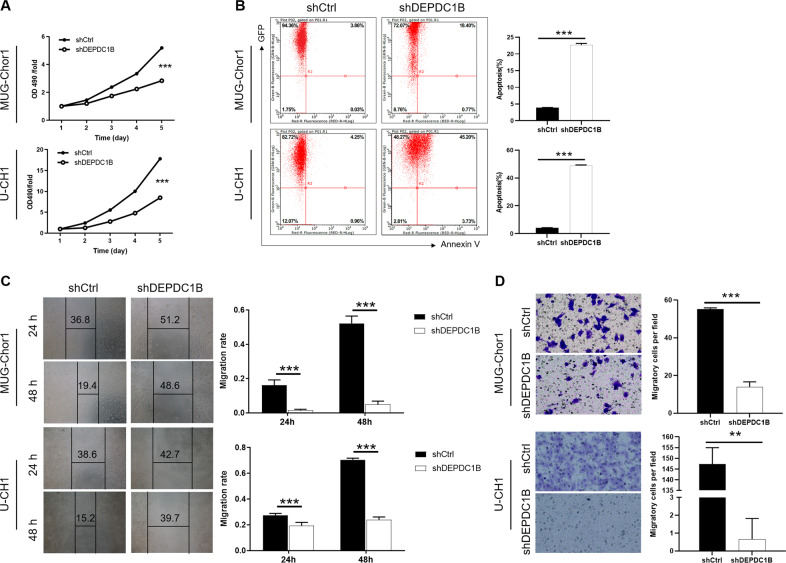
Knockdown of DEPDC1B inhibits cell proliferation and migration, promotes apoptosis in chordoma cells. **A** Cell proliferation of U-CH1 and MUG-Chor1 cells with or without knockdown of DEPDC1B was evaluated by MTT assay. **B** Flow cytometry analysis based on Annexin V-APC staining was utilized to detect cell apoptotic ratio for U-CH1 and MUG-Chor1 cells. **C**, **D** Cell migration of U-CH1 and MUG-Chor1 cells with or without knockdown of DEPDC1B was evaluated by Transwell assay (**D**) and wound-healing assay (**E**). The presented results were representative of experiments repeated at least three times. Data were represented as mean ± SD. **P < 0.01, ***P < 0.001.

First of all, WB results showed that the protein level of DEPDC1B was highly expressed in U-CH1 and MUG-Chor1 cells (Fig. S1A). Subsequently, the cells with knockdown of DEPDC1B were applied to detect the alterations of biological behavior. As illustrated in Fig. S1B, more than 80% of U-CH1 and MUG-Chor1 cells with GFP indicated the high infection efficiency. Moreover, the significant downregulation of the RNA (Fig. S1C) and protein (Fig. S1D) levels of DEPDC1B in U-CH1 and MUG-Chor1 cells consistently suggested that DEPDC1B was successfully knocked down. As a consequence, the effects of alteration of DEPDC1B expression on chordoma cells was investigated in vitro. The results of MTT detection showed that the OD490 value of shDEPDC1B group was remarkably lower than that of the shCtrl group (*P* < 0.001), which revealed that the downregulation of DEPDC1B led to the decrease of U-CH1 and MUG-Chor1 cells viability (Fig. 1A). Furthermore, the apoptosis percentage of shDEPDC1B group was significantly increased than that of shCtrl group (*P* < 0.001), indicating that the knockdown of DEPDC1B increased the apoptosis susceptibility of chordoma cells (Fig. 1B). Interestingly, the scratch distance of the shDEPDC1B group was obviously shorter than that of the shCtrl group between 0 and 48 h (*P* < 0.001) (Fig. 1C). Consistently, the migration fold change of shDEPDC1B group was indeed lower than that of shCtrl group (*P* < 0.001), which further verified that the downregulation of DEPDC1B expression can inhibit the ability of cell migration (Fig. 1D). Taken together, the above results demonstrated that the downregulation of DEPDC1B can inhibit the malignant behaviors of chordoma cells by weakening viability, enhancing apoptosis, and reducing migration.

### Knockdown of DEPDC1B regulates apoptosis-related factors and AKT, ERK, RHOA/ROCK cascades

**Fig. 2:**
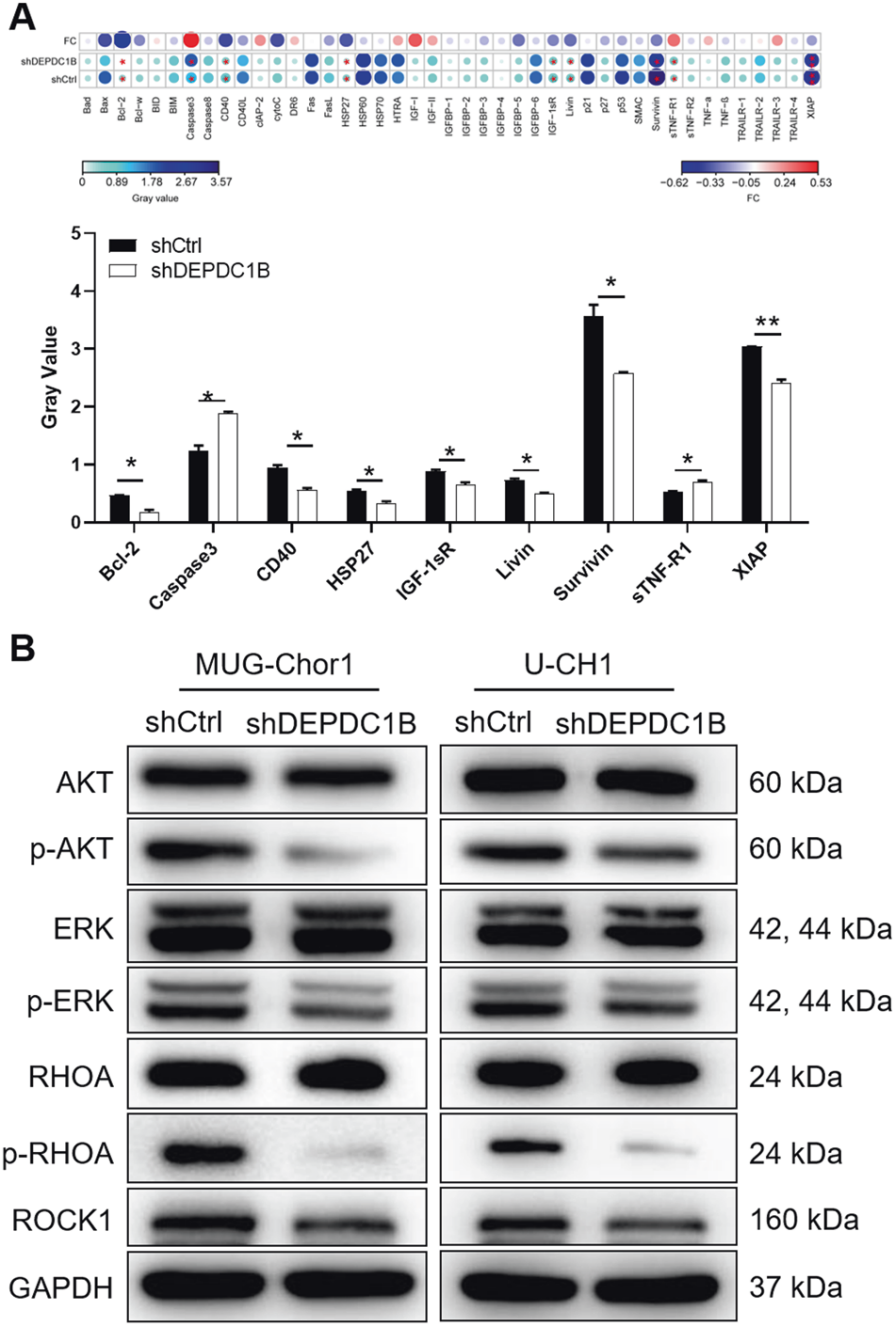
Knockdown of DEPDC1B regulates apoptosis-related factors and AKT, ERK, RHOA/ROCK cascades. **A** Human apoptosis antibody array analysis was performed in MUG-Chor1cells with or without DEPDC1B knockdown. **B** We tested the protein expression of AKT, p-AKT, ERK, p-ERK, RHOA, p-RHOA, ROCK1 after the expression of DEPDC1B decreased. Data was shown as mean ± SD. *P < 0.05.

Additionally, the effect of DEPDC1B on apoptosis-related factors was revealed by human apoptosis antibody array membrane. We found that knockdown of DEPDC1B resulted in downregulation of Bcl-2, CD40, HSP27, IGF-1sR, Livin, Survivin, XIAP and upregulation of Caspase3, sTNF-R1 in MUG-Chor1 cells (*P* < 0.05) (Fig. 2A). As DEPDC1B was previously described as involved in signaling by Rho-GTPases and by G-proteins [13, 14], the expression of MAPK/AKT and RHOA/ROCK cascades related proteins was explored. The results indicated that knockdown of DEPDC1B contributed to downregulation of p-AKT, p-ERK, p-RHOA, and ROCK1 expression (Fig. 2B). As a consequence, knockdown of DEPDC1B could regulate the apoptosis-related factors and AKT, ERK, RHOA/ROCK cascades.

### Knockdown of DEPDC1B suppresses tumor growth in mice

**Fig. 3:**
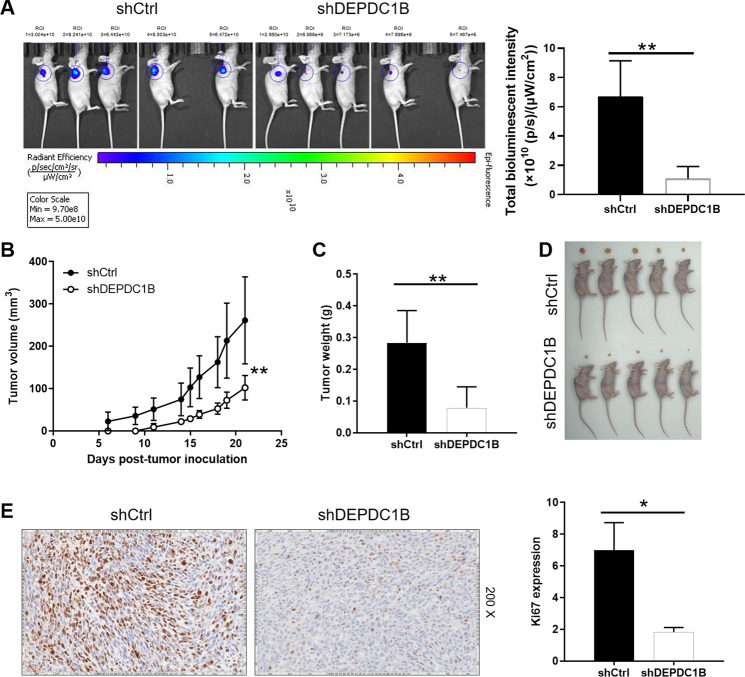
Knockdown of DEPDC1B inhibits tumor growth in mice xenograft models. **A** The total bioluminescent intensity of tumors in shCtrl group and shDEPDC1B group. **B** The volume of tumors in shCtrl group and shDEPDC1B group was measured after post-injection. **C** The average weight of tumors in shCtrl group and shDEPDC1B group. **D** Images of mice and tumors in shCtrl group and shDEPDC1B group. **E** The Ki67 staining of tumor tissues in shCtrl group and shDEPDC1B group. Data were represented as mean ± SD. *P < 0.05, **P < 0.01.

The effects of DEPDC1B on chordoma regulation was further elucidated by mouse xenograft model. The total bioluminescent intensity of mice was clearly presented, which reflected that the tumor load of shDEPDC1B group was obviously weaker than that of the control group (P < 0.01) (Fig. 3A). In addition, the size and volume of xenografts were measured and calculated throughout the animal experiments for 21 days, suggesting the significantly slower growth rate and smaller final tumor volume of shDEPDC1B group than the shCtrl group (P < 0.01) (Fig. 3B–D). Additionally, the results of immunohistochemical detection in the tumor tissues showed that the expression of Ki67 in shDEPDC1B group was lower than that in shCtrl group (P < 0.05) (Fig. 3E), indicating that downregulation of DEPDC1B can inhibit tumor proliferative activity in mice. Collectively, knockdown of DEPDC1B suppressed tumor growth of chordoma in vivo.

### DEPDC1B affects the BIRC5 ubiquitination through UBE2T in chordoma cells

**Fig. 4:**
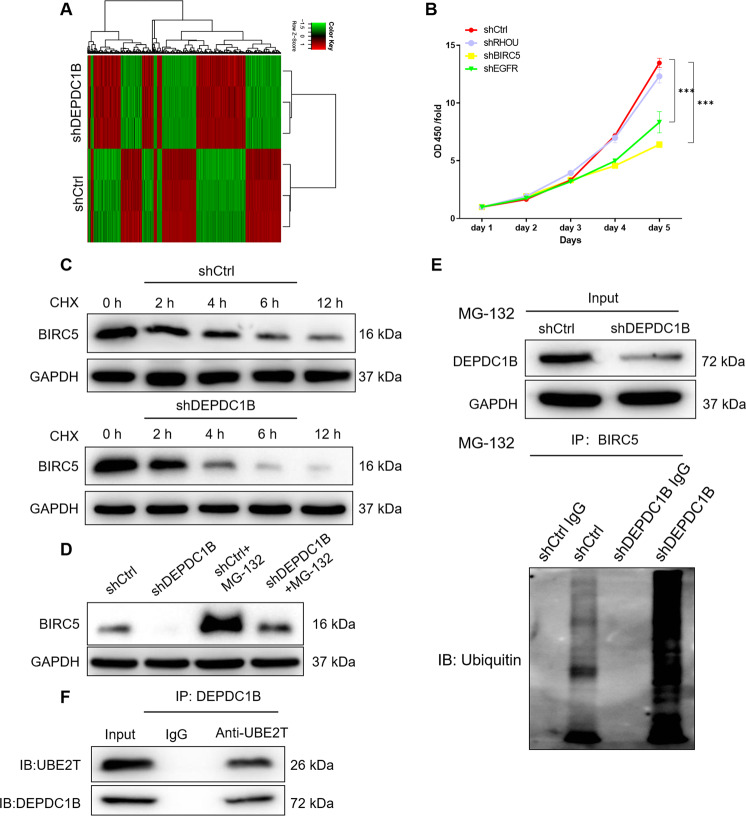
DEPDC1B affects the BIRC5 ubiquitination through UBE2T in chordoma cells. **A** The PrimeView Human Gene Expression Array was performed to identify the differentially expressed genes (DEGs) between shDEPDC1B and shCtrl groups of MUG-Chor1 cells. **B** The effects of knockdown of RHOU, BIRC5, and EGFR on proliferation of MuG-Chor1 cells were examined. **C** Protein levels of BIRC5 in MUG-Chor1 cells with or without DEPDC1B knockdown following 0.2 mg/mL CHX treatment for 0–12 h. **D** Levels of BIRC5 proteins in MUG-Chor1 cells with or without DEPDC1B knockdown following MG-132 treatment for indicated times (12 h). **E** The lysates of MUG-Chor1 cells were immunoprecipitated and WB was performed to examine the ubiquitination of BIRC5. **F** Co-IP analysis of interaction of DEPDC1B and UBE2T in MUG-Chor1 cells. The presented results were representative of experiments repeated at least three times. Data were represented as mean ± SD. ***P < 0.001.

The molecular mechanism of chordoma regulated by DEPDC1B was preliminarily explored. Firstly, knockdown of DEPDC1B resulted in DEGs in MUG-Chor1 cells, with 858 upregulated genes and 878 downregulated genes, which were presented in the hierarchical clustering (Fig. 4A). Furthermore, the significant enrichment of DEGs in canonical pathway as well as diseases and functions were investigated based on IPA, suggesting that ‘colorectal cancer metastasis signaling, Wnt/β-catenin signaling, Wnt/ca^+^ pathway’ (Fig. S2A) and cell proliferation, death and other functions (Fig. S2B) would be affected by DEPDC1B. Subsequently, the DEGs with the most significant alterations were selected by PCR (Fig. S2C) and WB (Fig. 2D). Figure S2E showed the interaction network based on IPA, in which DEPDC1B can affect BIRC5, EGFR, RHOU, and others. According to the results of protein interaction analysis (https://www.string-db.org/cgi/network?taskId=bVGGIYt5GuUj&sessionId=bVQ88ddIyQRY.), we found that DEPDC1B and BIRC5, EGFR, and RHOU both interact and regulate each other (Fig. S2F). Therefore, only these three genes were selected for subsequent experiments. Next, lentivirus was used to deliver shRNAs into MUG-Chor1 cells to knockdown these genes, respectively. As shown in the Fig. 4B, the inhibitory effect of shBIRC5 group on proliferation was the most significant compared with other groups (*P* < 0.001). BIRC5 was the most relevant to the proliferative phenotype of chordoma cells. As a consequence, BIRC5 was preliminarily identified as a downstream target of DEPDC1B in chordoma cells. On the other hand, upon the treatment of CHX (0.2 mg/mL, protein synthesis inhibitor), the protein stability of BIRC5 in shCtrl and shDEPDC1B chordoma cells was examined, indicating that DEPDC1B silencing induced a decrease of BIRC5 protein stability (Fig. 4C). Notably, the effects of DEPDC1B knockdown on BIRC5 protein stability could be partially eliminated by the treatment of MG-132 (20 μM), an inhibitor of proteasome (Fig. 4D), suggesting that DEPDC1B may regulate BIRC5 through ubiquitin-proteasome system (UPS) [28, 29]. Subsequently, we evaluated the regulation of DEPDC1B on ubiquitination of BIRC5 and the results indicated that DEPDC1B downregulation distinctly promotes BIRC5 ubiquitination (Fig. 4E). Considering that our previous study showed the regulation of BIRC5 ubiquitination by ubiquitin-conjugating enzyme E2T (UBE2T) (data not shown), we further explored the interaction between UBE2T and DEPDC1B. As shown in Fig. 4F, the direct interaction between DEPDC1B and UBE2T make us believe that DEPDC1B may influence UBE2T-mediated ubiquitination of BIRC5 through interacting UBE2T.

### Simultaneous downregulation of BIRC5 and DEPDC1B exacerbates the inhibitory effects of chordoma

**Fig. 5:**
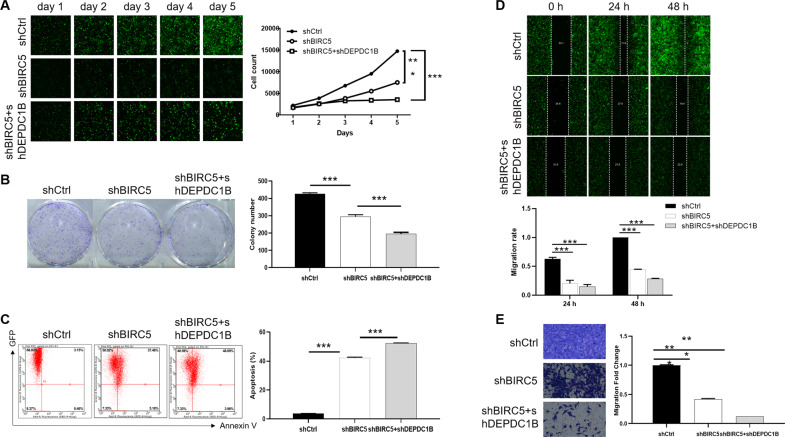
Knockdown of BIRC5 deepens the effects on chordoma cells by DEPDC1B knockdown. After lentivirus shCtrl, shBIRC5 or shBIRC5 + shDEPDC1B infected with MUG-Chor1 cells, they were subjected to the detection of proliferation (**A**), clone formation (**B**), apoptosis (**C**) and migration (**D**, **E**). The presented results were representative of experiments repeated at least three times. Data were represented as mean ± SD. *P < 0.05, **P < 0.01, ***P < 0.001.

The biological behavior of BIRC5 and DEPDC1B in MUG-Chor1 was elucidated by loss-of-function assays. Firstly, the protein level of BIRC5 was highly expressed in U-CH1 and MUG-Chor1 cells, which was similar to DEPDC1B (Fig. S3A). Secondly, the protein of shBIRC5-1 group was downregulated most significantly, which was used to construct cell model of knockdown of BIRC5 (Fig. S3B). Moreover, MUG-Chor1 cells not only had high infection efficiency (Fig. S3C), but also the protein level of BIRC5 and DEPDC1B was significantly downregulated (Figure S3D), which indicated that BIRC5 and DEPDC1B was knocked down successfully. Notably, shBIRC5 was downregulation of BIRC5, shBIRC5 + shDEPDC1B was simultaneous downregulation of BIRC5 and DEPDC1B in MUG-Chor1 cells. As a result, the results showed a significant inhibition in the progression of BIRC5-knocked-down MUG-Chor1 cells, such as inhibited proliferation (*P* < 0.001) (Fig. 5A), decreased clones (*P* < 0.001) (Fig. 5B), enhanced apoptosis (*P* < 0.001) (Fig. 5C) and hindered migration (Fig. 5D, E). Besides, simultaneous downregulation of BIRC5 and DEPDC1B could exacerbate the inhibitory effects of chordoma cell progression, which was characterized by inhibiting proliferation (*P* < 0.001, fold change = −3.3) (Fig. 5A), reducing clone formation (*P* < 0.001, fold change = −7.0) (Fig. 5B), stimulating apoptosis (*P* < 0.001, fold change = 14.1) (Fig. 5C) and repressing migration (*P* < 0.001, >70%) (Fig. 4D, E).

### BIRC5 overexpression attenuates the inhibitory effects of DEPDC1B knockdown in chordoma cells

**Fig. 6:**
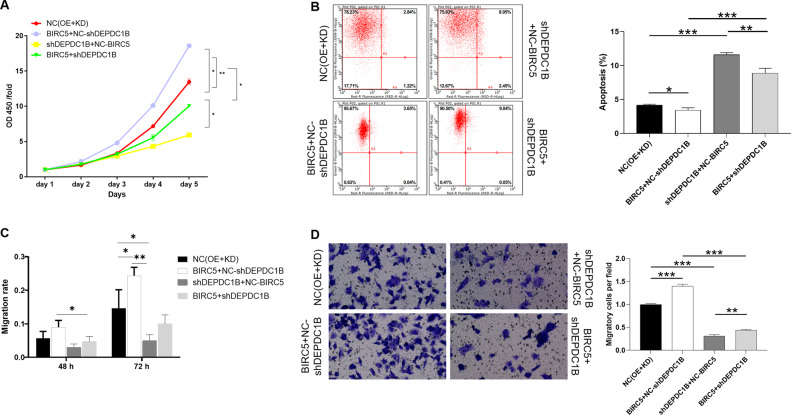
Overexpression of BIRC5 reduces the inhibitory of DEPDC1B knockdown on the malignant behaviors of chordoma cells. After lentivirus NC(OE+KD), BIRC5+NC-shDEPDC1B, shDEPDC1B+NC-BIRC5, or BIRC5+sh DEPDC1B infected with MUG-Chor1 cells, they were subjected to the detection of proliferation (**A**), apoptosis (**B**), and migration (**C**, **D**). The presented results were representative of experiments repeated at least three times. Data were represented as mean ± SD. *P < 0.05, **P < 0.01, ***P < 0.001.

To fully demonstrate the effects of DEPDC1B and BIRC5 in chordoma cells, the functional recovery assays was conducted. Accordingly, the MUG-Chor1 cells with simultaneously upregulated BIRC5 and downregulated DEPDC1B (BIRC5 + sh DEPDC1B) were established (Fig. S4A–C). Notably, NC(OE+KD) group was the MUG-Chor1 cells infected with negative control lentivirus; BIRC5+NC-shDEPDC1B group was the overexpression of BIRC5; shDEPDC1B+NC-BIRC5 group was the cells with low expression of DEPDC1B. As illustrated in Fig. 6A, compared with NC (OE+KD) group, cell proliferation was weakest in shDEPDC1B+NC-BIRC5 group (*P* < 0.05), and strongest in BIRC5+NC-shDEPDC1B group (*P* < 0.01); The BIRC5+shDEPDC1B group can attenuate the inhibitory effect of cell proliferation (*P* < 0.05). Meanwhile, apoptosis was the weakest in the BIRC5+NC-shDEPDC1B group (*P* < 0.05). On the contrary, shDEPDC1B+NC-BIRC5 group of apoptosis was the strongest (*P* < 0.001) (Fig. 6B). The apoptosis of BIRC5+shDEPDC1B group was higher than that of BIRC5+NC-shDEPDC1B group (*P* < 0.001), but weaker than shDEPDC1B+NC-BIRC5 group (*P* < 0.01). There was no doubt that the cell migration rate was the highest in the BIRC5+NC-shDEPDC1B group and the lowest in the shDEPDC1B+NC-BIRC5 group compared with NC(OE+KD) group (Fig. 6C, S5A). The BIRC5+shDEPDC1B group can reduce the inhibitory effect of shDEPDC1B+NC-BIRC5 group in chordoma cells (*P* < 0.05). Unsurprisingly, the results of the Transwell experiment were consistent with these trends (Fig. 6C). Therefore, overexpression of BIRC5 can reduce the inhibitory effect of DEPDC1B knockdown on the malignant behaviors of chordoma cells.

## Discussion

In this study, the unique role and potential molecular mechanism of DEPDC1B in chordoma were recognized. Specifically, knockdown of DEPDC1B inhibits the malignant behavior of chordoma cells in vitro, such as reduction of proliferation, induction of apoptosis, and inhibition of migration. As expected, downregulation of DEPDC1B suppressed growth of chordoma in vivo. In particular, the knockdown of DEPDC1B resulted in not only a significant upregulation of Caspase3, sTNF-R1, but also downregulation of Bcl-2, CD40, HSP27, IGF-1sR, XIAP, Livin, Survivin in chordoma cells. In addition, apoptosis involves both internal and external pathways, in which the endogenous pathway is mediated by an anti-apoptotic protein Bcl-2 (B-cell lymphoma-2) that integrates death and survival signals [30]. CD40 interacts with CD40 ligand (CD40L) to regulate apoptosis according to different membrane localization [31]. HSP27 is a molecular chaperone with the ability to interact with a large number of proteins, which could regulate apoptosis by interacting with components involved in Caspase activation and apoptosis [32]. Moreover, XIAP, an X-linked inhibitor of apoptosis, exerts a strong inhibitory effect on apoptosis depending on its unique ability to bind Caspases [33]. As members of the inhibitors of apoptosis (IAP) family, Livin and Survivin are abnormally expressed in the progression of cancers, which could inhibit Caspases and prevents cell death [34, 35]. Not surprisingly, the molecular mechanism of chordoma cell apoptosis induced by DEPDC1B knockdown is complex, which required the participation of a series of apoptosis-related factors.

Importantly, apoptosis repeat-containing 5 (BIRC5) was considered to be the downstream target of DEPDC1B involved in the progression of chordoma. Previous study reported that BIRC5 is a member of IAP family, which is a mitotic spindle checkpoint gene and encodes Survivin protein. Additionally, BIRC5 acts as a bifunctional regulator of apoptosis inhibition and cell cycle progression [36, 37]. Moreover, BIRC5 is highly expressed in tumors, including cancer cells and tumor stem cells, whose expression is associated with the differentiation, proliferation, invasion and metastasis of tumor cells [38–41]. In recent years, considerable evidence suggested that abnormal expression of BIRC5 is involved in the progression of various cancers, including lung, breast, colon, pancreatic, and prostate cancers [42–46]. Although abnormal expression of BIRC5 is significantly associated with tumor progression, the exact role and molecular mechanism of BIRC5 in chordoma have not been determined. The present study clarified that DEPDC1B affected the ubiquitination of BIRC5 through UBE2T. Ubiquitination is a widespread post-translational modification that mediates the localization, metabolism, function, regulation, and degradation of proteins in cells [47]. The UPS is composed of ubiquitin, ubiquitin-activating enzyme E1, ubiquitin-conjugating enzyme E2, ubiquitin ligase E3, proteasome and its substrates. Studies have shown that UPS plays a central role in regulating protein levels and activities, cell cycle, gene expression, response to oxidative stress, cell survival, proliferation and apoptosis, which is closely related to the onset of cancers and cardiovascular diseases [48]. Moreover, UBE2T is a member of the E2 family in the UPS [49]. Recently, Yin et al, reported that UBE2T promoted radiation resistance of non-small cell lung cancer through ubiquitin-mediated FOXO1 degradation [50]. Our study clarified that DEPDC1B affected the ubiquitination of BIRC5 through UBE2T, leading to dysregulation of gene expression. Furthermore, simultaneous downregulation of BIRC5 and DEPDC1B may exacerbate the inhibitory effects of chordoma cells. Moreover, overexpression of BIRC5 can reduce the inhibitory effect of DEPDC1B knockdown on the malignant behaviors of chordoma cells.

In conclusion, DEPDC1B affected the ubiquitination of BIRC5 through UBE2T. Simultaneous downregulation of BIRC5 and DEPDC1B may exacerbate the inhibitory effects of chordoma. Moreover, BIRC5 overexpression reduced the inhibitory effects of DEPDC1B knockdown in chordoma cells. DEPDC1B regulates the progression of human chordoma through UBE2T-mediated ubiquitination of BIRC5, which may be a promising candidate target in molecular therapy of chordoma patients.

## Supplementary information

Table S1

Table S2

Supplementary figure legends

Figure S1

Figure S2

Figure S3

Figure S4

Figure S5
