# Correction to: DEPDC1B regulates the progression of human chordoma through UBE2T-mediated ubiquitination of BIRC5

**DOI:** 10.1038/s41419-022-05095-y

**Published:** 2022-07-21

**Authors:** Liang Wang, Liang Tang, Ruijun Xu, Junpeng Ma, Kaibing Tian, Yanbin Liu, Yanghu Lu, Zhen Wu, Xiaodong Zhu

**Affiliations:** 1 Department of Neurosurgery, Beijing Tiantan Hospital, Capital Medical University, No. 119 Nansihuan Xilu, Beijing, 100070 China; 2 Department of Orthopaedic Surgery, Tongren Hospital, Shanghai Jiao Tong University School of Medicine, No. 1111 Xianxia Road, Shanghai, 200336 China; 3 Department of Orthopaedic Surgery, Renji Hospital, Shanghai Jiao Tong University School of Medicine, No. 2000 Jiangyue Road, Shanghai, 200127 China

**Keywords:** CNS cancer, Oncogenes

Correction to: *Cell Death & Disease* 10.1038/s41419-021-04026-7, published online 30 July 2021

The original version of this article contained a mistake in the PDF of the article. The corrected statement for annotation 4 in the PDF should read the same as the online version: These authors contributed equally: Liang Wang, Liang Tang. The original article has been corrected.

