# Supplementary material for: DEPDC1B regulates the progression of human chordoma through UBE2T-mediated ubiquitination of BIRC5
Source: Cell Death Dis. 2021 Jul 30;12(8):753. doi: 10.1038/s41419-021-04026-7 (PMC8324777; doi:10.1038/s41419-021-04026-7)
Supplement: Supplementary file 1 — Table S1 [file 41419_2021_4026_MOESM1_ESM.docx]

Primers used in qPCR

| Gene | Forward primer sequence (5’-3’) | Reverse primer sequence (5’-3’) |
| --- | --- | --- |
| DEPDC1B | CTGAAGTGACCCGCAAACAAA | CTGGTGGGAGATCATTCCATTC |
| GAPDH | TGACTTCAACAGCGACACCCA | CACCCTGTTGCTGTAGCCAAA |
| MAPK1 | GACTGGACGTGCTCAGACAT | CCTCCAAACGGCTCAAAGGA |
| FOS | CAGACTACGAGGCGTCATCC | TCTGCGGGTGAGTGGTAGTA |
| MYC | TGCTGCCAAGAGGGTCAAGT | GCTCCGTTTTAGCTCGTTCC |
| RHOU | CGCCTCCTACATCGAGTGTT | GACTTCTTTGGCTGTTGCTGA |
| BIRC5 | TCTCAAGGACCACCGCATCT | TTTGCATGGGGTCGTCATCT |
| FGFR1 | TGCCCGCCAACAAAACA | AATCTTGCTCCCATTCACCTC |
| PIM1 | TTTTCTTCAGGCAGAGGGTCT | GGAGGTGGATCTCAGCAGTTT |
| JUN | TGCCTCCAAGTGCCGAAAA | TAAGCTGTGCCACCTGTTCC |
| PIK3CD | GTGAACGGCAGGCATGAGTA | AGGATGGAGGAGGAATGGAC |
| TGFBR2 | GTGCCAACAACATCAACCACA | GCCTTATAGACCTCAGCAAAGC |
| EGFR | ATGAGGACATAACCAGCCACC | AGGCACGAGTAACAAGCTCAC |
| GNG12 | ATGTCCAGCAAAACAGCAAGC | CCTATCAGCAAAGGGTCACTCC |
| CCND1 | ATGTCCAGCAAAACAGCAAGC | CCTATCAGCAAAGGGTCACTCC |
| FZD1 | CTCTTCGTGTACCTGTTTATCGG | TGGTGCCATCGTGCTTCAT |
| RRAS2 | GAGGCATCAGCAAAGATTAGGA | GGTTCTGGTGAAGGAGGACATT |
| MAPK9 | CTCTGCGTCACCCATACATCA | TCTTTCTTCCAACTGGGCATC |
| ADCY1 | ATGACCTGCGAGGACGATGA | TTCTAAGAGGCGGCTGATGTA |
| TGFB2 | CCCACTTTCTACAGACCCTACTTC | AATCCGTTGTTCAGGCACTC |
| CTNNB1 | TGCGTTCTCCTCAGATGGTG | TGGGAAAGGTTATGCAAGGTC |
