# Supplementary material for: DEPDC1B regulates the progression of human chordoma through UBE2T-mediated ubiquitination of BIRC5
Source: Cell Death Dis. 2021 Jul 30;12(8):753. doi: 10.1038/s41419-021-04026-7 (PMC8324777; doi:10.1038/s41419-021-04026-7)
Supplement: Supplementary file 2 — Table S2 [file 41419_2021_4026_MOESM2_ESM.docx]

Antibodies used in western blotting and IHC

| Primary antibodies | Dilution in WB | Source species | Company | Catalog No. |
| --- | --- | --- | --- | --- |
| DEPDC1B | 1:1000 | Rabbit | Abcam | ab124182 |
| AKT | 1:3000 | Rabbit | Proteintech | 10176-2-AP |
| p-AKT | 1:2000 | Mouse | Proteintech | 66444-1-Ig |
| ERK | 1:2000 | Rabbit | R&D | AF1051 |
| p-ERK | 1:1000 | Rabbit | R&D | AF1018 |
| RHOA | 1:500 | Mouse | Santa Cruz | sc-418 |
| p-RHOA | 1:500 | Rabbit | Bioss | bs-5330R |
| ROCK1 | 1:1000 | Goat | Santa Cruz | sc-6055 |
| BIRC5 | 1:1000 | Rabbit | Abcam | ab469 |
| EGFR | 1:1000 | Rabbit | Abcam | ab52894 |
| RHOU | 1:1000 | Rabbit | Abcam | ab80315 |
| UBE2T | 1:50/1:2000 | Rabbit | Proteintech | 10105-2-AP |
| Ubiquitin | 1:750 | Rabbit | Proteintech | 10201-2-AP |
| GAPDH | 1:3000 | Rabbit | Bioworld | AP0063 |
| Primary antibodies | Dilution in IHC | Source species | Company | Catalog No. |
| Ki67 | 1:200 |  | Abcam | ab16667 |
| Secondary antibody | Dilution |  | Company | Catalog No. |
| HRP Goat Anti-Rabbit IgG (WB) | 1:3000 | Rabbit | Beyotime | A0208 |
| HRP Goat Anti- Mouse IgG (WB) | 1:3000 | Mouse | Beyotime | A0216 |
| HRP Donkey Anti-Goat | 1:3000 | Goat | Beyotime | A0181 |
| HRP Goat Anti-Rabbit IgG (IHC) | 1:400 |  | Abcam | ab6721 |
