# Supplementary material for: DEPDC1B regulates the progression of human chordoma through UBE2T-mediated ubiquitination of BIRC5
Source: Cell Death Dis. 2021 Jul 30;12(8):753. doi: 10.1038/s41419-021-04026-7 (PMC8324777; doi:10.1038/s41419-021-04026-7)
Supplement: Supplementary file 3 — Supplementary figure legends [file 41419_2021_4026_MOESM3_ESM.docx]

**Figure S1.** (A) The protein expression of DEPDC1B in human chordoma cells U-CH1 and MUG-Chor1 was measured by WB. (B) The infection efficiencies of shCtrl and shDEPDC1B in MUG-Chor1 cells were evaluated through observing the fluorescence of GFP on lentivirus vector. (C, D) The knockdown efficiencies of DEPDC1B in MUG-Chor1 cells was evaluated at levels of mRNA (C) and protein (D). The presented results were representative of experiments repeated at least three times.

**Figure S2.** (A) The enrichment of the DEGs in canonical signaling pathways was analyzed by IPA. (B) The enrichment of the DEGs in IPA disease and function was analyzed by IPA. (C) The mRNA level of DEGs after knockdown of DEPDC1B was measured by qPCR. (D) WB were used to detect the expression of several selected DEGs in MUG-Chor1 cells with or without DEPDC1B knockdown. (E) The interaction network based on IPA. (F) Protein interaction was analyzed through https://www.string-db.org/cgi/network?taskId=bVGGIYt5GuUj&sessionId=bVQ88ddIyQRY.

**Figure S3.** (A) The protein expression of BIRC5 in human chordoma cells U-CH1 and MUG-Chor1. (B) The knockdown efficiencies of 3 shRNAs designed for BIRC5 knockdown were evaluated by WB. (C) The infection efficiency of shCtrl, shBIRC5, shBIRC5+shDEPDC1B in MUG-Chor1 cells were evaluated through observing the GFP on lentivirus vector. (D) The knockdown efficiency of shCtrl, shBIRC5, shDEPDC1B+shBIRC5 in MUG-Chor1 cells were accessed by WB. The presented results were representative of experiments repeated at least three times.

**Figure S4.** After lentivirus NC(OE+KD), BIRC5+NC-shDEPDC1B, shDEPDC1B+NC-BIRC5 or BIRC5+shDEPDC1B infected with MUG-Chor1 cells, the mRNA level of BIRC5 (A) or DEPDC1B (B) evaluated by qPCR. (C) The protein expression of BIRC5 or DEPDC1B was evaluated by WB in NC(OE+KD), BIRC5+NC-shDEPDC1B, shDEPDC1B+NC-BIRC5 or BIRC5+shDEPDC1B groups. The presented results were representative of experiments repeated at least three times. Data was represented as mean ± SD. *P < 0.05, **P < 0.01, ***P < 0.001.

**Figure S5.** (A) Scratch pictures of MUG-Chor1 cells in NC(OE+KD), BIRC5+NC-shDEPDC1B, shDEPDC1B+NC-BIRC5 or BIRC5+shDEPDC1B groups during 0 h, 48 h and 72 h. The presented results were representative of experiments repeated at least three times.
