# Supplementary figures and images for: DEPDC1B regulates the progression of human chordoma through UBE2T-mediated ubiquitination of BIRC5
Source: Cell Death Dis. 2021 Jul 30;12(8):753. doi: 10.1038/s41419-021-04026-7 (PMC8324777; doi:10.1038/s41419-021-04026-7)

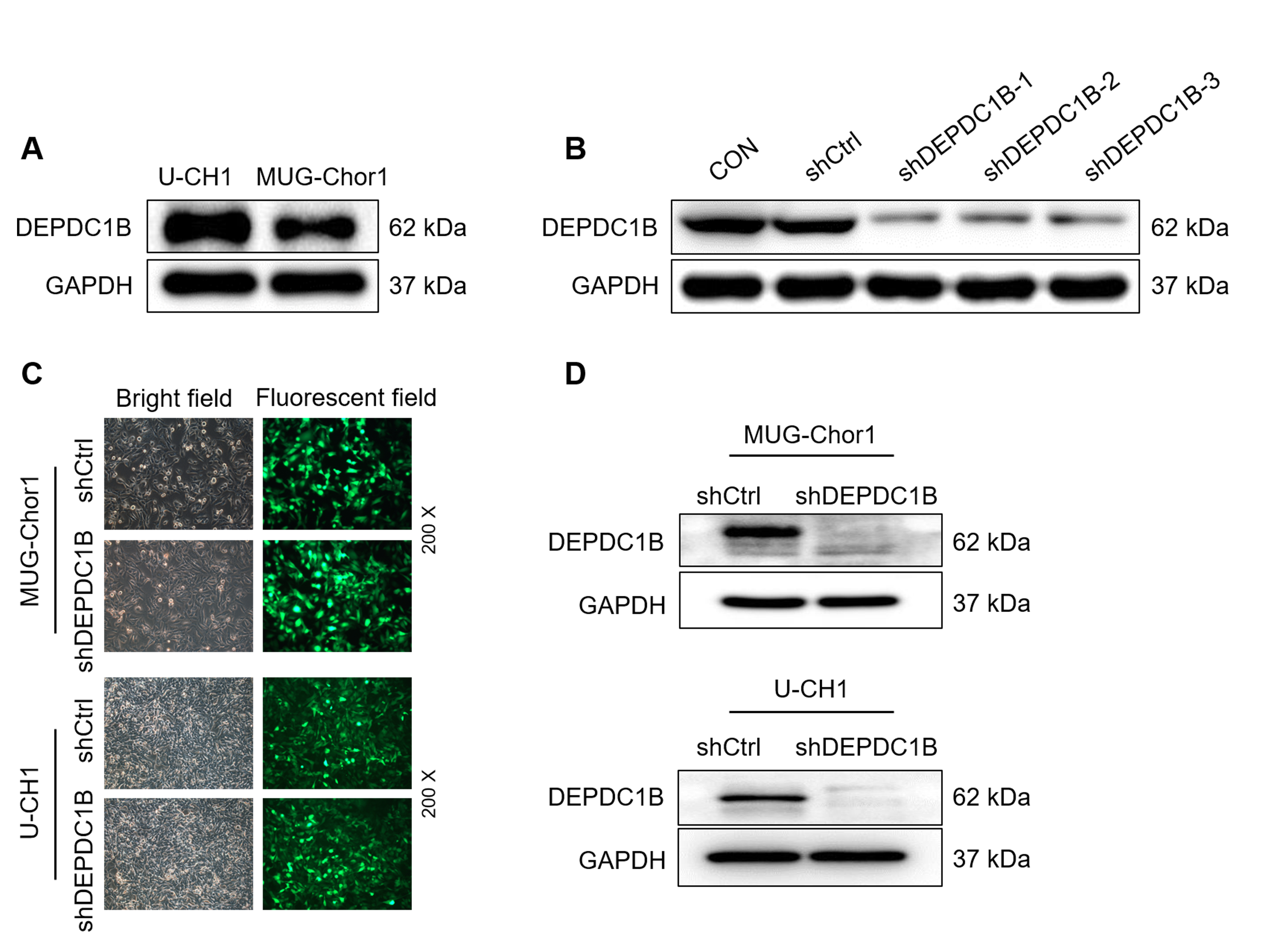

Supplement: Supplementary file 4 — Figure S1 [file 41419_2021_4026_MOESM4_ESM.tif]

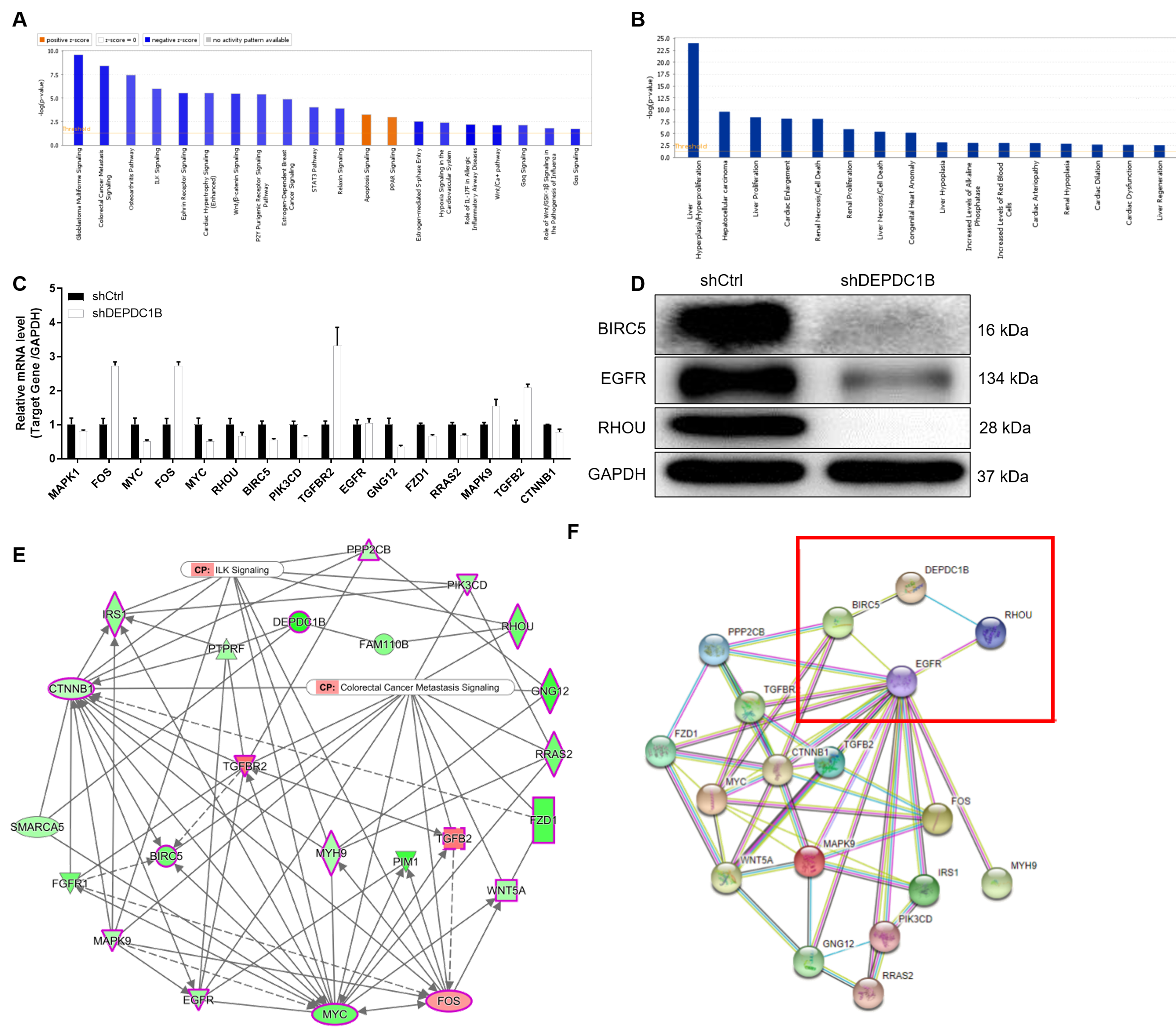

Supplement: Supplementary file 5 — Figure S2 [file 41419_2021_4026_MOESM5_ESM.tif]

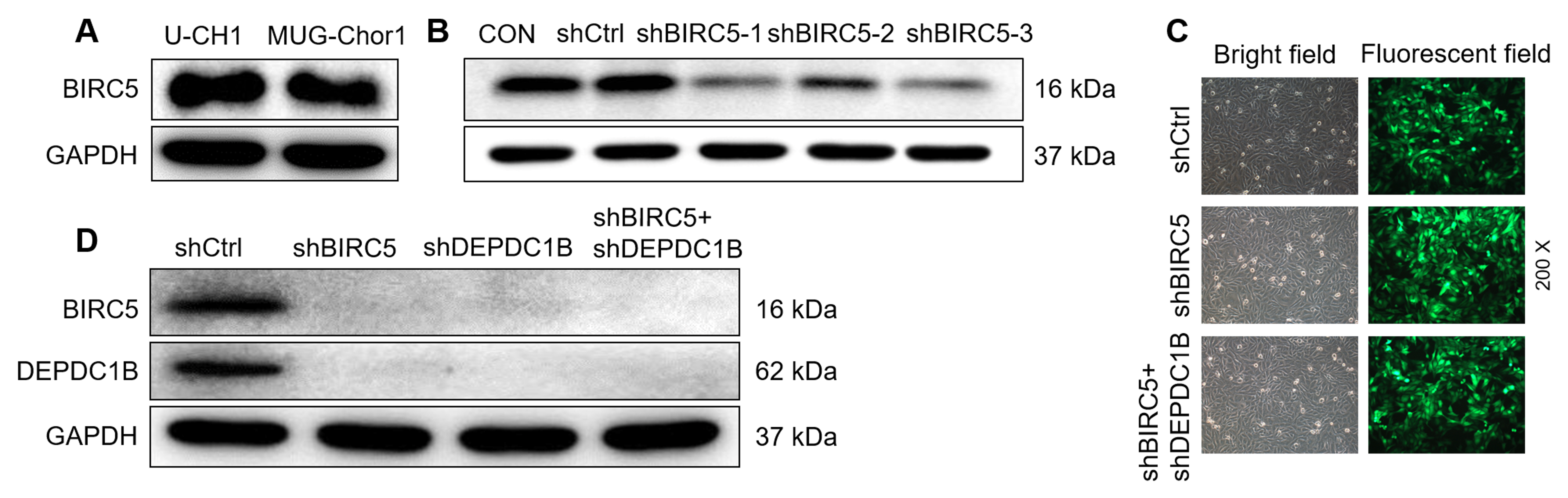

Supplement: Supplementary file 6 — Figure S3 [file 41419_2021_4026_MOESM6_ESM.tif]

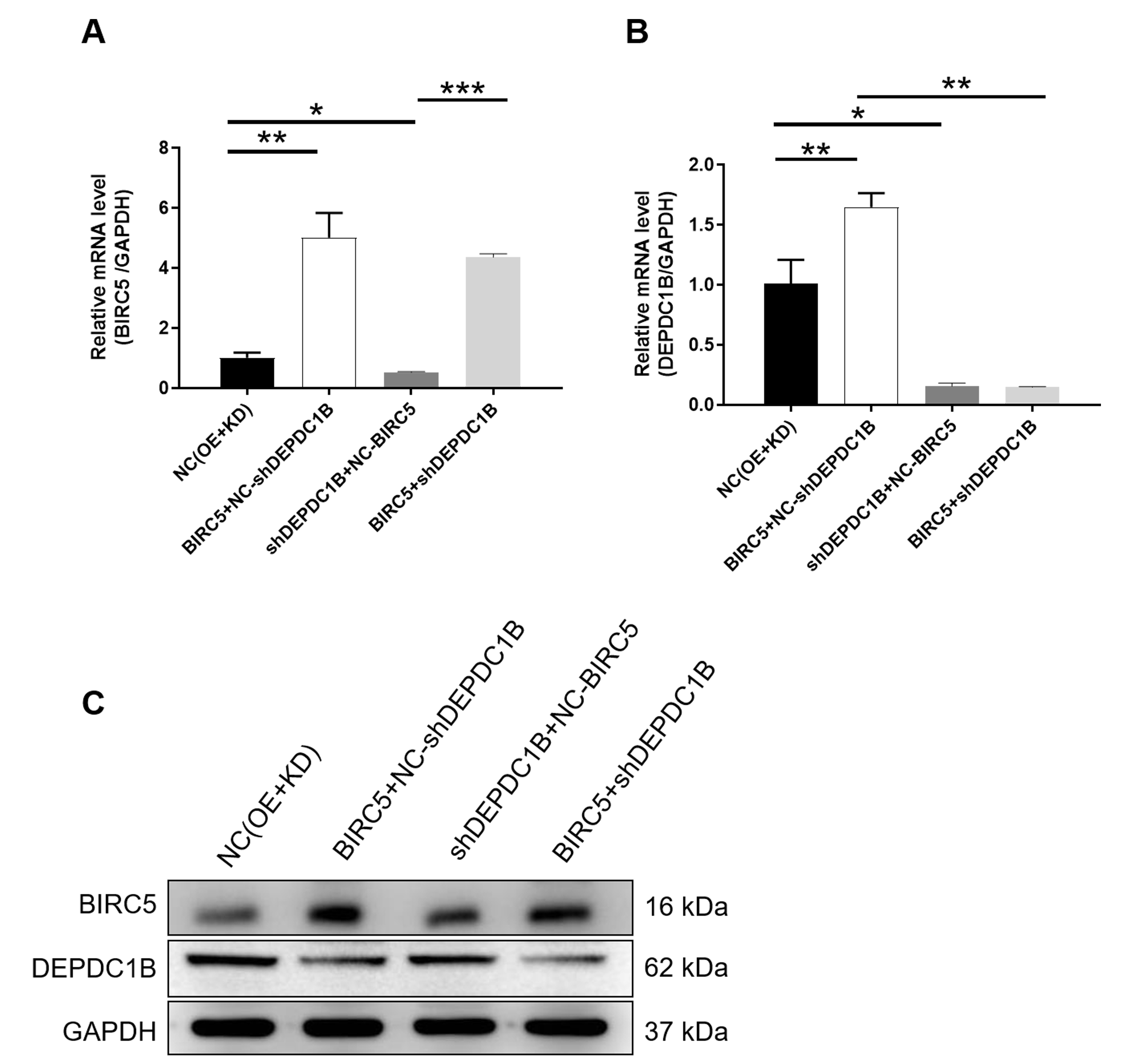

Supplement: Supplementary file 7 — Figure S4 [file 41419_2021_4026_MOESM7_ESM.tif]

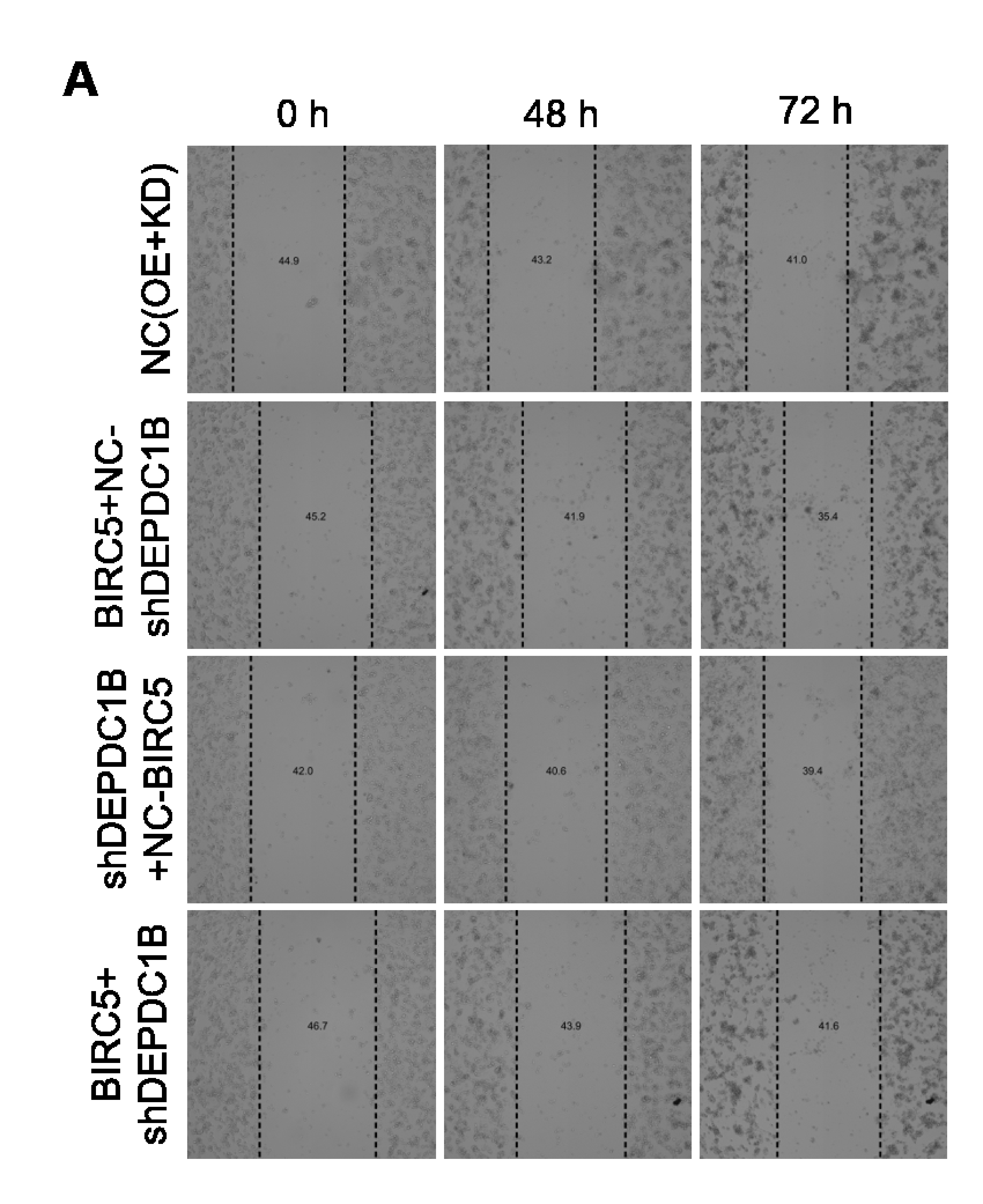

Supplement: Supplementary file 8 — Figure S5 [file 41419_2021_4026_MOESM8_ESM.tif]
